# Is Aducanumab treatment developed to prevent progression of Alzheimer's disease cost-effective in Turkey?

**DOI:** 10.1186/s12962-023-00463-7

**Published:** 2023-08-09

**Authors:** Vahit Yigit, Selin Kalender, Iskender Cetinturk

**Affiliations:** 1 Faculty of Economics and Administrative Sciences, Health Management Department, Suleyman Demirel University, 32200 Isparta, Turkey; 2 Health Social Sciences Institute, Health Economics Doctorate Program, Suleyman Demirel University, 32200 Isparta, Turkey

**Keywords:** Aducanumab, Alzheimer’s disease, Cost-effectiveness analysis, Markov model

## Abstract

**Background:**

Alzheimer's Disease (AD), the most common type of dementia, is a chronic, progressive, and neurodegenerative brain discomfort that causes the be damage to brain cells. Although there is no definitive treatment for AD, various drug treatments are used to reduce and control the symptoms of the disease. Developed for the treatment of mild-stage Alzheimer's patients, Aducanumab is the only drug approved by the Food and Drug Administration (FDA) in the past two decades. However, the cost is very high and, in many countries, Aducanumab has not been approved due to insufficient clinical efficacy and lack of evidence yet. This study aims to analyze the cost-effectiveness of Aducanumab, which was developed for the treatment of mild-stage AD, from the patient's perspective.

**Methods:**

In the study, the Markov model was developed to determine the cost-effectiveness of Aducanumab compared to Standard of Care (SoC) therapy over a 5-year horizon. Cost and effectiveness data were taken from the literature. In the study, the discount rate was determined as 6%. The results were presented as the incremental cost-effectiveness ratio (ICER), which represents the cost per quality-adjusted life years (QALY). The results were retested with a one-way and probabilistic sensitivity analysis (PSA) due to possible uncertainties in the research parameters. The results were presented with the tornado diagram and the scatter plots.

**Results:**

With the Markov model, the total costs of Aducanumab and SoC treatments over a 5-year horizon were found to be 98.068 $ and 21.292 $, respectively. Aducanumab treatment had an incremental gain of 0.64 QALY and an incremental cost of 76.776 $ compared to the SoC treatment. The ICER value, which shows the additional cost per QALY of Aducanumab, was 119.408 $/QALY. As a result of the study, it was determined that Aducanumab was not cost-effective when compared to SoC treatment. Sensitivity analysis results showed stability against uncertainties. Aducanumab was confirmed not to be cost-effective with its current price and potential clinical benefit.

**Conclusion:**

The result of the research is considered important in terms of providing evidence-based information on the cost-effectiveness of Aducanumab in Turkey. However, further, research is needed to evaluate Aducanumab's clinical efficacy and cost-effectiveness.

**Supplementary Information:**

The online version contains supplementary material available at 10.1186/s12962-023-00463-7.

## Background

Alzheimer's Disease (AD), the most common type of dementia, is a chronic, progressive, and neurodegenerative disorder caused by damage to nerve cells in the brain [1]. The disease is characterized by the accumulation of neurofibrillary tangles in intracellular areas (intracellular) and plaques containing β-amyloid (Aβ) in extracellular areas in the brain tissue. These accumulating plaques are considered the basic pathophysiology of AD and are used in the differential diagnosis of the disease. Aβ accumulation disrupts neural communication between cells, damage to cells, and death of many cells. As a result, the brain becomes dysfunctional [2–5]. The process, which starts with difficulty in remembering, cognitive dysfunction, changes in routine behaviors, and learning difficulties in Alzheimer's patients [1, 6], progresses to speech-walking problems and irreversible loss of basic functions such as chewing-swallowing [2]. The progression of the disease is clinically defined in three stages mild (early), moderate (middle,) and severe (late) AD [7].

Although there is no definitive treatment for AD, current treatments (cholinesterase inhibitors and memantine) aim to reduce the disease’s symptoms and are widely used [8]. The effect of these treatments on reducing or controlling patients' cognitive and behavioral symptoms has been clinically proven. However, progressive degeneration of brain cells continues even when patients are under control with current treatments. Therefore, new treatment forms related to the pathophysiology of AD are being investigated [5]. Especially Disease-Modifying Therapies (DMTs) that target the mechanisms underlying AD and slow/stop the progression of the disease when used in the early stages gain importance [9–11].

Adanucumab is the first disease-modifying drug developed for the treatment of mild-stage AD. It is also the only treatment approved for AD by the Food and Drug Administration (FDA) in the past two decades [12]. Double-blind, randomized, and placebo-controlled studies have shown that Adanucumab promotes the clearance of Aβ plaques accumulated in the brain [13] and significantly reduces plaque accumulation. Unlike existing treatments, Adanucumab has a mechanism that targets and influences the basic pathophysiology of AD. For this reason, the approval process for Aducanumab has been accelerated by the FDA so that patients can access the drug earlier [14]. However, clinical trials for Aducanumab are ongoing. The clinical benefit of Aducanumab is expected to be proven as a result of the Phase IV clinical trial, which is expected to be completed in 2030. Otherwise, it is possible to withdraw the approval given by the FDA. Results from clinical trials to date for Aducanumab have been inconsistent and uncertain. Aducanumab has not yet been approved in many countries, especially in EU countries, due to the lack of enough evidence regarding its clinical efficacy [11, 13, 15]. In addition to the uncertainty in the clinical efficacy of Aducanumab, also the high list price is considered to be a worrying situation [16]. Similar to other countries, a formal approval process for Aducunumab has not yet been initiated in Turkey. Therefore, the drug is not within the scope of reimbursement. However, special permission has been granted by the Turkey Ministry of Health for the first time to use Aducanumab under routine control in a patient in 2021. The cost of the Aducanumab was paid by the patient. [17]. In this context the study, it was aimed to evaluate the cost-effectiveness of Aducanumab from a patient perspective in Turkey. The study was conducted according to the principles of the Turkish version of Consolidated Health Economic Evaluation Reporting Standards (CHEERS) [18].

## Methods

The average life expectancy of Alzheimer's patients is 8–10 years from diagnosis. In the early stage of the disease, the average life expectancy is 2 years. [19]. Aducanumab, used in the early stage of AD, is a treatment aimed at slowing the progression of the disease [20]. In economic evaluation studies, the time horizon should be of sufficient length to cover all costs and effects associated with the treatment [21]. The time horizon of the study was determined as 5 years under the assumption that Aducanumab used in the early stage would slow down the progression of the disease and prolong the transition to the moderate stage. A Markov state transition model was developed to evaluate the cost-effectiveness of Aducanumab from a patient perspective compared to Standard of Care (SoC) treatment alone in the study.

In the study, SoC therapy was determined as a combination of symptomatic drug therapies and supportive care. Symptomatic drug therapies are the medical treatment with agents (cholinesterase inhibitors, memantine, etc.) developed to reduce and slow down the symptoms of AD [22]. Supportive care is the non-pharmacological care therapy given by patient relatives, social care centers and health professionals in order to improve the quality of life of Alzheimer's patients [23].

**Fig. 1:**
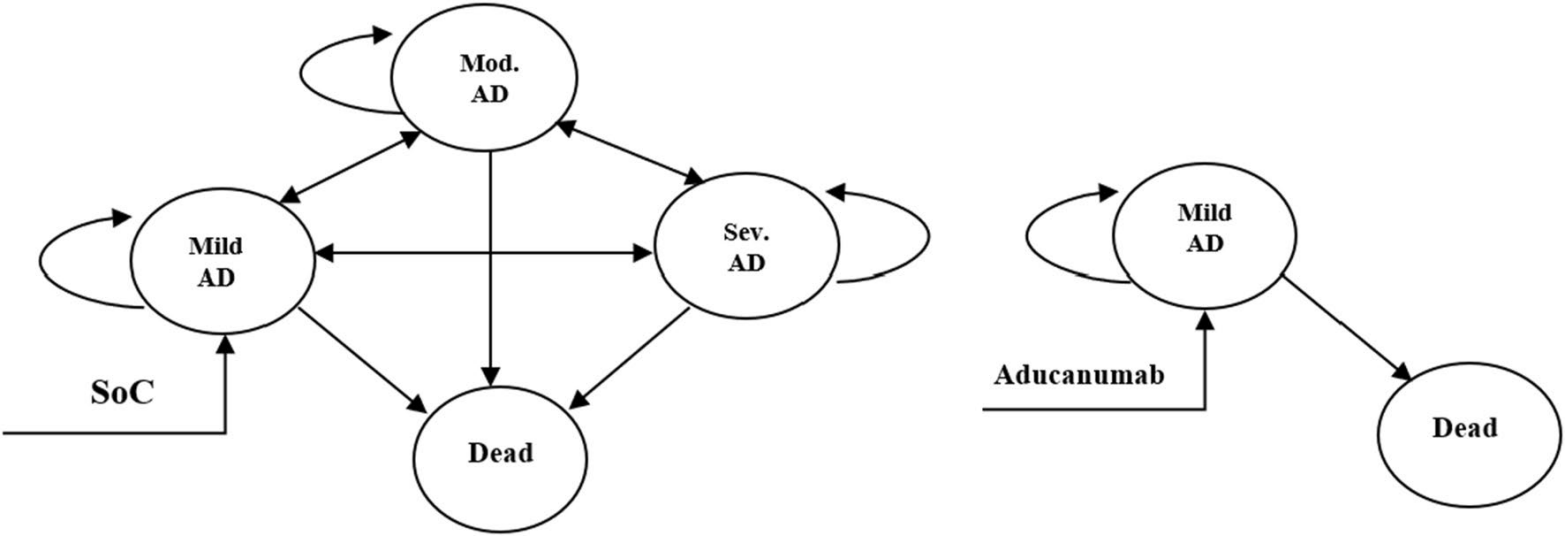
Markov transition model

AD is relatively difficult to diagnose at the preclinical stage when mild cognitive impairment occurs [24]. Newly developed treatments also often target the early stage of the disease [9–11]. There are various cost-effectiveness studies in the literature in which the progression of AD is modeled from the preclinical stage [25–28]. However, the approach in which disease progression is modeled in three stages as early, moderate, and severe [29] is widely used in cost-effectiveness analysis studies [30]. This study aimed to evaluate the economic impact of Aducanumab treatment targeting the early stage of the disease. For this reason, in accordance with the purpose of the research, it was preferred to use a model from the literature in which AD progresses from the early stage [16]. The model simulates a hypothetical cohort of 1000 male and female Alzheimer's patients. The age of onset of the cohort was accepted as 65, since AD is common in people over the age of 65 [31]. The model consists of 4 health states depending on the clinical progression of the disease (See Fig. 1). These are mild, moderate, and severe stages of AD and death. Patients enter the model from the mild AD stage. In the model, patients transition from mild AD to moderate and severe AD, and then in the death state. There are no calculated transition probabilities for AD in Turkey. For this reason, the transition probabilities used in the model (see Table 1) were obtained from the literature [32].

**Table 1:** Transition probabilities matrix

	Mild AD	Moderate AD	Severe AD	Death
Mild AD	0.774	0.158	0.013	0.055
Moderate AD	0.070	0.501	0.214	0.215
Severe AD	0.001	0.027	0.492	0.480
Death	0.000	0.000	0.000	1.000

Reference: [32]

The model was established under the best-case scenario assumption that Aducanumab completely arrests disease progression in mild AD. Aducanumab was considered clinically efficacy in this scenario. Therefore, it was assumed that patients treated with Aducunumab did not progress to moderate to severe AD stages. Clinical efficacy data of Aducanumab obtained from EMERGE and ENGAGE Phase III clinical trial results are inconsistent [33]. Therefore, in the study, it was assumed that a hypothetical DMT used in the treatment of AD has high clinical efficacy and reduces disease progression by 40% (Relative Risk-RR = 0.6). RR was included in the transition probabilities matrix of Aducanumab. In the transition probabilities matrix, the probability of patients receiving Aducunumab treatment remaining in the mild AD state was increased considering the clinical effectiveness of the treatment in reducing disease progression (RR = 0.6). Briefly, the probability of staying in the same state was increased by taking into account the percentage of patients whose disease did not progress as a result of the clinical efficacy of Aducanumab. Then, the probability of transitioning from the mild state to the death state was decreased by considering the probability of staying in the same state. Thus, the sum of the transition probabilities in the model has remained as 1. It was assumed that SoC treatment did not have any reducing effect on the progression of AD and the RR value was accepted 1. Therefore, no correction was made in the transition probability matrix of patients receiving SoC treatment. The cost [17, 34] and effectiveness data [35] used for baseline and scenario analyses in the study were obtained from the literature. An ethical committee report was not necessary for this study which used secondary data derived from the literature.

The costs assigned to each Markov state for SoC therapy are taken from an article made in Turkey [34]. In this article, annual health care costs (direct + indirect) were calculated according to the stages of AD for 2017. Since the costs of medical care (symptomatic drugs, medical treatment, etc.) and the supportive care costs (non-pharmacological) are taken into account in the SoC therapy, the total costs in the article was used. The effective selling rate of the Central Bank of Turkey was used to convert costs from Turkish Lira (TL) to United States Dollar (USD) [36]. For the cost of Aducanumab, a paper containing the cost of treatment with Aducanumab for a patient in Turkey was taken as a reference [17]. A discount rate of 6% was used for costs and outcomes other than the first year [37]. 1The threshold value was determined as three times the GDP [38] by the World Health Organization (WHO) recommendation. Cost and outcome data and all other research parameters (see Table 2) were placed in the TreeAge Healthcare Pro program to create the Markov model (Additional file 1: Figure S1) and the model was run. The results were presented as the incremental cost-effectiveness ratio (ICER), which represents the cost per quality-adjusted life year (QALY). Under the assumption that the research results contain uncertainty, a one-way sensitivity analysis was performed. The parameters determined are costs, utility values, transition probabilities, and discount rate. The uncertainty around the base case results was tested using 95% confidence intervals (CI) in the sensitivity analysis. The results were presented with a Tornado diagram. To test the uncertainty in all parameters at the same time, probabilistic sensitivity analysis (PSA) was performed with a second-order Monte Carlo simulation. In the PSA, beta distributions for utility values, transition probabilities; gamma distributions for costs [39] were assumed. Analysis was done with 1000 simulations (repeats) and 500 sample exchanges. Results were shown on scatter plots showing QALY difference versus cost difference. The acceptability of Aducanumab for different threshold values was also tested in the PSA. The results were presented on the cost-effectiveness acceptability curve [32].

**Table 2:** Basic research parameters

Research parameters	Aducanumab	SoC	References	Disturubution
Costs-Annual ($)	Mild $22,000*	Mild $3,140^#^ Moderate $9,460^#^ Severe $16.956^#^	*[17] ^#^[34]	Gamma
Effectiveness data	Mild 0.73	Mild 0.73 Moderate 0.69 Severe 0.27	[35]	Beta
Model and transition probabilities	See Fig. 1, Table 1	See Fig. 1, Table 1	[32]	Beta
Number of cohorts	1000	1000		NA
Clinical efficacy data	RR = 0.6	–	Assumption	NA
Horizon	5 year	5 year		NA
Discount Rate	6%	6%	[37]	(3–9%)
Threshold Value^+^	$28,617	$28,617	[38]	NA
Perspective	Patient	Patient		–

*NA*: Not assessed

^+^Calculated based on 2022 GDP

*Aducanumab annual cost

#SoC annual cost

## Results

In the results section below, the results of cost-effectiveness analysis, one-way sensitivity analysis, and PSA were presented, respectively.

### Cost-effectiveness analysis results

**Fig. 2:**
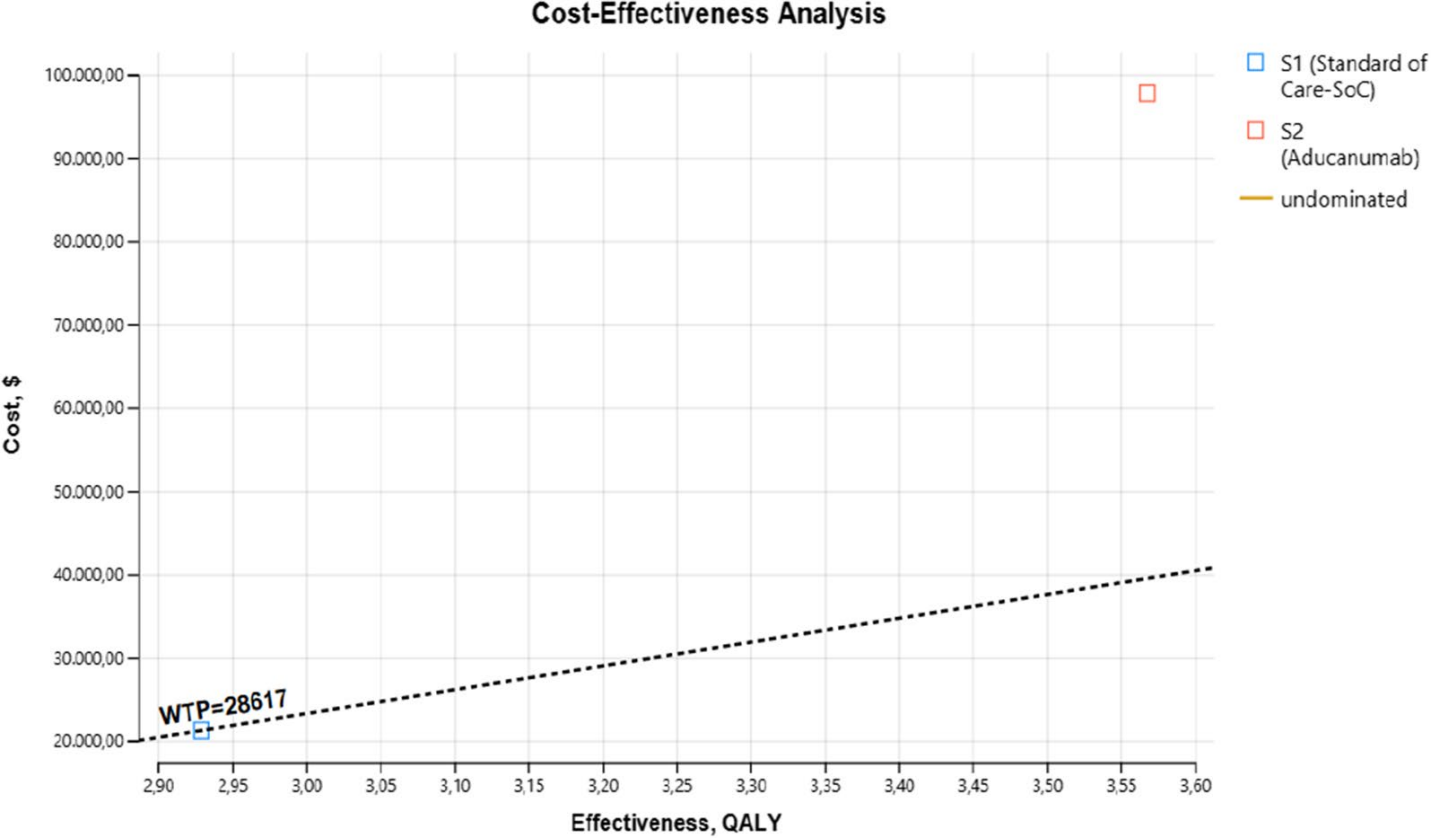
Presentation of results in cost-effectiveness plane

In the study, the total costs and total QALYs of Aducanumab and SoC treatments were calculated during the horizon determined as 5 years. A 6% discount was applied to results other than the first year. According to the calculation result, the cumulative QALY sum of treatment with SoC was 2.94 over the 5 years, and the cumulative QALY total of treatment with Aducanumab is 3.58. The 5-year total cost calculated for SoC treatment was $21,292.16. The 5-year total cost of aducanumab treatment was $98,068.49. The distribution of treatments on the cost-effectiveness plane is given in Fig. 2.

When Fig. 2 is examined, it is seen that the treatment with Aducanumab at a threshold value of $ 28,617 is well above the threshold value. This result reveals that SoC is the most cost-effective treatment approach compared to Aducanumab under the limited healthcare budget. In the cost-effectiveness analysis, the ICER value showing the cost per incremental QALY of Aducunumab was calculated and compared with the SoC. The cost-effectiveness analysis results are presented in detail in Table 3.

**Table 3:** Cost effectiveness analysis results

Treatment options	Total cost ($)	Total effectiveness (QALY)	Incremental cost ($)	Incremental effectiveness (QALY)	ICER ($/QALY)	Decision
SoC	21.292,16	2.94	–	–	–	Dominant
Aducanumab	98.068,49	3.58	76.776,33	0.64	119.482,80	

Compared to SoC, Aducaumab has higher effectiveness (3.58) and provides an incremental 0.64 QALY gain. However, the overall cost of Aducanumab (98,068.49) is also higher. Aducanumab has an incremental cost of $76,776.33 compared to the SoC. The calculated ICER for aducanumab is $119,482.80. This value is well above the threshold value. Therefore, Aducanumab cannot be considered a cost-effective treatment at a threshold value of $28,617. However, when evaluated with the current clinical efficacy results, it is seen that the effect of Aducanumab on the health outcomes (quality of life) of the patients is higher compared to the SoC. Thus, it is anticipated that treatment with Aducanumab will be considered cost-effective if the annual cost of treatment is reduced.

**Fig. 3:**
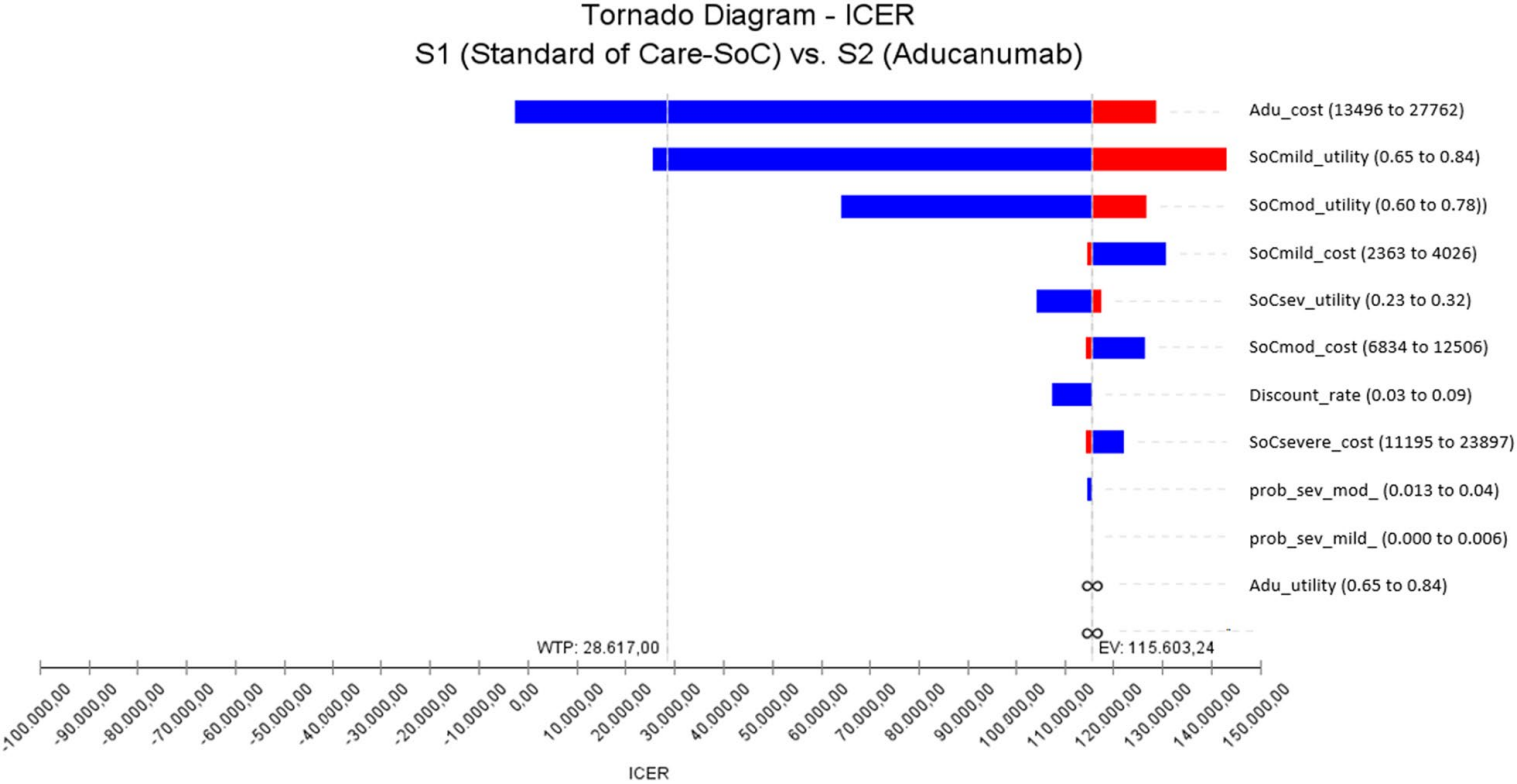
Tornado diagram showing the result of one-way sensitivity analysis

### One-way sensitivity analysis results

Below is the tornado diagram in which the result of the one-way sensitivity analysis performed to test the uncertainty of the analysis results are presented (see Fig. 3).

One-way sensitivity analysis showed a stable result in all parameter changes between the determined lower and upper limits. It was found that possible uncertainties in the parameters did not have any effect on the analysis results and Aducanumab was not cost-effective. The expected value of ICER for Aducunaumab remained at $115,603.24. However, in the analysis results, it was determined that the first two parameters with the greatest effect on ICER were the annual treatment cost of Aducanumab and the quality value of the life of patients with mild AD. Therefore, for Aducanumab to be a cost-effective option, it is thought that the annual treatment cost should be less than $22,000.

### Probabilistic sensitivity analysis results

PSA (Monte Carlo simulation) was performed with hypothetical 1000 replicates and 500 samples to simultaneously evaluate the uncertainty of the analysis results. Incremental costs per QALY were recalculated for Aducanumab and SoC in the analysis. The scatter chart showing the analysis results is presented below.

**Fig. 4:**
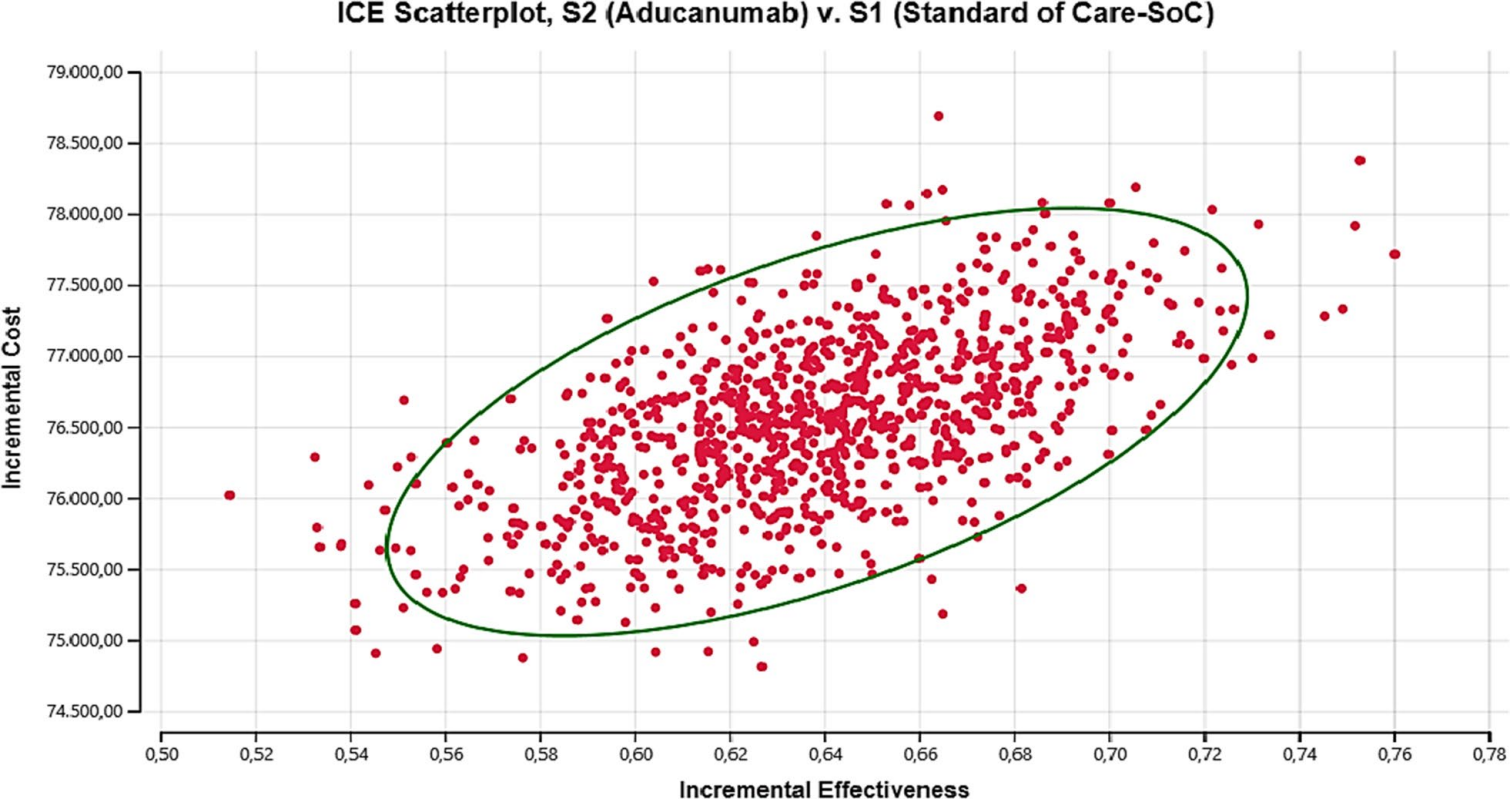
Scatterplot of all simulated ICER values of aducanumab

Figure 4 shows the probabilistic distribution of ICER points corresponding to the comparison of Aducanumab with SoC. When Fig. 4 is examined, it is seen that 100% of the points are well above the limit determined for the threshold value ($28,617). At a threshold value of $28,617 per QALY, Aducanumab was not found to be cost-effective in 100% of all comparisons. Aducanumab remained well above the established threshold of cost-effectiveness due to being a very expensive treatment compared to existing treatments.

It is known that the calculated ICER values exceed the traditionally determined threshold values due to the relatively high costs of newly developed treatments. Discussions and studies continue how the threshold value should be determined for new treatments [40]. However, although they are above the threshold value in cost-effectiveness comparisons, the results calculated for these treatments are valuable for decision-makers. Therefore, the acceptability of Aducanumab was retested for different thresholds on the cost-effectiveness acceptability curve (Additional file 1: Figure S2).

Aducanumab is not cost-effective in 100% of comparisons at the threshold of $0–97,298. However, it was found that the probability of cost-effectiveness of Aducanumab increased to 0.2% with the increase of the threshold value to $103.021. Similarly, was determined that the probability of Aducanumab being cost-effectively increased to 2% with the increase of the threshold value to $108,745. Finally, when the threshold is raised to $114,468, the probability of Aducanumab being cost-effective rises to 18.2%. According to the results obtained from the acceptability curve, it was determined that the probability of cost-effectiveness of Aducanumab increases if the threshold value for Turkey is higher than $100,000.

## Discussion and conclusion

In this study, the cost-effectiveness of Aducanumab, which is not yet within the scope of reimbursement in Turkey, was evaluated with the Markov model. The clinical efficacy of Aducanumab on the progression of AD was assumed to be high. However, research results revealed that Aducanumab is not cost-effective compared to SoC over a 5-year horizon for $22,000 per year. The results were retested with one-way and PSA due to possible uncertainties in the research parameters. Sensitivity analysis results showed stability against uncertainties. Aducanumab was confirmed not to be cost-effective with its current price and potential clinical benefit. However, with a threshold value increase to $114,418, the probability of Aducanumab being cost-effectively increased to 18.2%, also. Although the model of this study was created with limited data obtained from the literature, the results obtained provide a perspective on the potential clinical effectiveness and economic value of Aducanumab in developing countries such as Turkey. In this context, it also contributes to the economic evaluation literature on Aducanumab. In addition, the cost-effectiveness results of Aducanumab provide evidence-based information to the reimbursement agency and health policymakers in Turkey.

There are several studies in the literature evaluating the cost-effectiveness of Aducanumab. Sinha and Barocas [13] evaluated the cost-effectiveness of Aducanumab compared to SoC treatment from a healthcare system perspective in the USA with the Markov model. The horizon was determined as 5 years in the model. In the study was found that compared to SoC treatment, Aducanumab cost $383,080 per QALY and was not cost-effective. In the threshold value analysis, was estimated to be cost-effective of Aducanumab if the annual cost of $56,000 was reduced to $22,820. Another study was conducted in the USA by Synnott et al. [16]. The study it was used the Markov model. The study is based on the health system and societal perspective. The horizon was determined as a lifetime in the model. The cost-effectiveness of Aducanumab was compared with supportive care treatment alone. It was noted that for aducanumab to be cost-effective at the threshold of $100,000–150,000, its annual price must be reduced by an average of $2,950 to $5,950. A study similar to that study of Synnott et al. was conducted in the USA by Whittington et al. [41]. The study, it was used the Markov model. The horizon was determined as a lifetime in the model. The cost-effectiveness of Aducanumab was compared with supportive care treatment alone also in this study. It has been stated that for Aducanumab to be considered cost-effective at the widely accepted threshold, its annual price should average between $2,950 and $8,360. A similar study evaluating the cost-effectiveness of Aducanumab was conducted in the USA by Ross et al. [42]. The cost-effectiveness of Aducanumab used for the treatment of mild-stage AD was compared with SoC. The study, in which an analytical decision model was used, was based on the health system and social perspective. The horizon was determined as a lifetime in the model. As a result of the study, it was determined that Aducanumab is not a cost-effective treatment at its current price, but can be cost-effective if the annual price drops below $3,000. In studies evaluating the cost-effectiveness of Aducanumab in the literature, it has been found that the drug is not cost-effective at its current list price. Although made with limited data from the literature, the results of the cost-effectiveness studies provide information on the potential economic impact of Aducanumab under uncertain clinical outcomes.

Since it is a newly developed treatment, the long-term clinical efficacy of Adunucumab in patients is not yet known. This situation is seen as worrying due to the less frequent follow-up of patients under routine care. In addition, the treatment has the potential for serious side effects. Also, it is unclear whether the treatment provides sufficient clinical benefit in the face of possible side effects, according to the results of the current clinical trial [14]. However, there are studies in the literature to evaluate the long-term clinical benefit of Aducanumab based on current clinical research results. The clinical benefit of long-term use of Aducanumab in patients with mild AD was evaluated based on EMERGE clinical efficacy data by Herring et al. [43]. The study, it was used the Markov model. The horizon was determined as a lifetime in the model. The clinical benefit of aducanumab was compared with the alone SoC. In the study, was found that Aducanumab had an incremental 0.65 QALY gain compared to patients treated with SoC over the lifetime horizon. However, it was stated by the researchers that more research is needed to confirm the long-term clinical benefit of Aducanumab.

The major criticism of Aducanumab in the literature is the lack of sufficient evidence for its clinical efficacy despite the very high cost of the treatment. However, despite uncertain results regarding the clinical benefit of Aducanumab, a treatment based on the pathophysiology of AD has been developed for the first time. This development is promising in terms of controlling the progression of the disease. However, due to the uncertainty in the clinical trial results of Aducanumab, concerns about its approval processes remain. It is thought that if the uncertainty regarding the clinical effectiveness of the treatment is eliminated as a result of ongoing clinical studies, the registration studies of the drug will be accelerated and approved in many countries. However, the current list price determined for Aducanumab is too high. When it comes to approval of the drug in Turkey, it is foreseen that the price should be reduced to be included in the scope of reimbursement. Aging and health problems due to old age are seen as global public health problems and are handled with a public health approach. Therefore, if the clinical efficacy of Aducanumab is proven, all mild-stage Alzheimer's patients should have equal access to available treatment. Thus, there is a need to develop global drug policies to reduce the price of Aducanumab. In the face of the possible decrease in the price of the drug, it is predicted that Aducunumab will fall below the threshold value in cost-effectiveness comparisons and will become a cost-effective treatment option. Similarly, Aducanumab, used in the treatment of patients with mild AD, is predicted to be a cost-effective treatment if it is proven to have higher clinical efficacy (more than 40%) as a result of the Phase IV clinical trial.

However, the annual costs of current treatments for AD are less (less than $500) compared to Aducanumab. If Aducanumab treatment proves to be no more effective than existing treatments, it is not possible to persuade funders and payers to pay for Aducanumab at its current price. Similar to the results of other studies in the literature, also this study the current price of Aducanumab and its cost per QALY were found to be well above the cost-effectiveness threshold. Under these conditions, it does not seem possible to obtain a reimbursement list of Aducanumab in Turkey. However, in this study, the threshold value was traditionally determined according to the WHO recommendation.

It is known that the limits of traditionally determined threshold values are low and these threshold values remain low for newly developed drugs. Therefore, there is a widespread opinion that levels of the threshold values should be raised. Or it is stated that a threshold value should be determined to reflect the economic value of the possible benefits of newly developed drugs. In addition, instead of applying the traditional threshold value with strict rules, it is recommended that each country determine a threshold value by its economic conditions. There is no official threshold value announced for Turkey. In this context, a formal threshold value needs to be determined. Considering that drug prices are affected by the increase in the exchange rate, is recommended that the threshold value per QALY be determined as foreign currency indexed in Turkey.

The elderly population is increasing rapidly around the world. Turkey is among the fast aging countries. The elderly population has increased by 24% in the last 5 years. It is predicted that the proportion of the elderly population, which is 9.7% by 2022, will increase to 12.3% in 2030 and 16.3% in 2040 [44]. AD, the most common type of dementia, is among the most common chronic health problems among the elderly population. In parallel with the increase in the elderly population, also the prevalence of dementia is increasing rapidly [34]. It was stated that 55 million people worldwide have dementia in 2021. It is estimated that this number will increase to 78 million in 2030 and 139 million in 2050. It is reported that the majority of the increase will occur in low and middle-income countries. The total cost of dementia, which was $818 million in 2015, is expected to rise to $2.8 trillion in 2030 [45, 46]. It is reported that there are between 600 thousand and 1 million Alzheimer's patients in Turkey in 2021. It is estimated that this number will increase unpredictably in 30–40 years [47]. Under the assumption that half of current Alzheimer's patients have mild stage Alzheimer's patients [48] the annual treatment cost of Aducanumab in Turkey is estimated to will be between $6.5 billion and $11 billion. It does not seem possible to cover this cost with the current budget of the reimbursement institution in Turkey.

In the guidelines developed for economic evaluation studies, the discount rate is frequently determined as 3% [49]. However, the 3% discount rate is not compatible with the economic context of low- and middle-income countries. It is stated that this ratio will lead to a systematic bias toward overestimating the future costs and health effects of interventions [37]. For this reason, in this study, the threshold value was determined as 6% in contrast to the widespread use of 3%. However, due to the differences in economic growth and the high inflationary environment, it is thought that there is a need to adopt the discount rate specific to Turkey.

## Limitations

This study should be evaluated considering that it contains certain limitations. The investigational model was created under the assumption that Aducanumab provides clinical benefit in the treatment of mild AD. The clinical efficacy data used in the model were taken from the literature because the clinical efficacy results obtained from the Phase III study of Aducanumab were inconsistent and uncertain. In addition, cost, effectiveness data, and transition probabilities were obtained from the literature. Due to limitations in the data, the results from the study cannot be definitively concluded that Aducanumab is not cost-effective. In this context, it is recommended to renew the analysis results with the current clinical efficacy data obtained when the ongoing phase IV clinical trial for Aducanumab is concluded.

### Supplementary Information

**Additional file 1: Figure S1.** Creation of Markov Model in Treeage Program. **Figure S2.** Acceptability of Aducanumab at Different Thresholds.

## Data Availability

The data and material this research will be made available from the corresponding author, following a reasonable submitted request.

## Abbreviations

Aβ: β-Amyloid
AD: Alzheimer’s disease
DMTs: Disease-Modifying therapies
FDA: Food and Drug Administration
GDP: Gross-domestic product
ICER: Incremental cost-utility ratio
QALY: Quality-adjusted life years
RR: Relative risk
SoC: Standard of care
TL: Turksih Liras
USD: United States Dollar
WHO: World Health Organization

## Footnotes

1 This study was presented as an oral presentation to the 7. International Health Sciences and Management Conference on 17 June 2022.
