# Supplementary material for: Is Aducanumab treatment developed to prevent progression of Alzheimer's disease cost-effective in Turkey?
Source: Cost Eff Resour Alloc. 2023 Aug 9;21:51. doi: 10.1186/s12962-023-00463-7 (PMC10410945; doi:10.1186/s12962-023-00463-7)

**Supplement 1**

**Figure 1.** Creation of Markov Model in Treeage Program


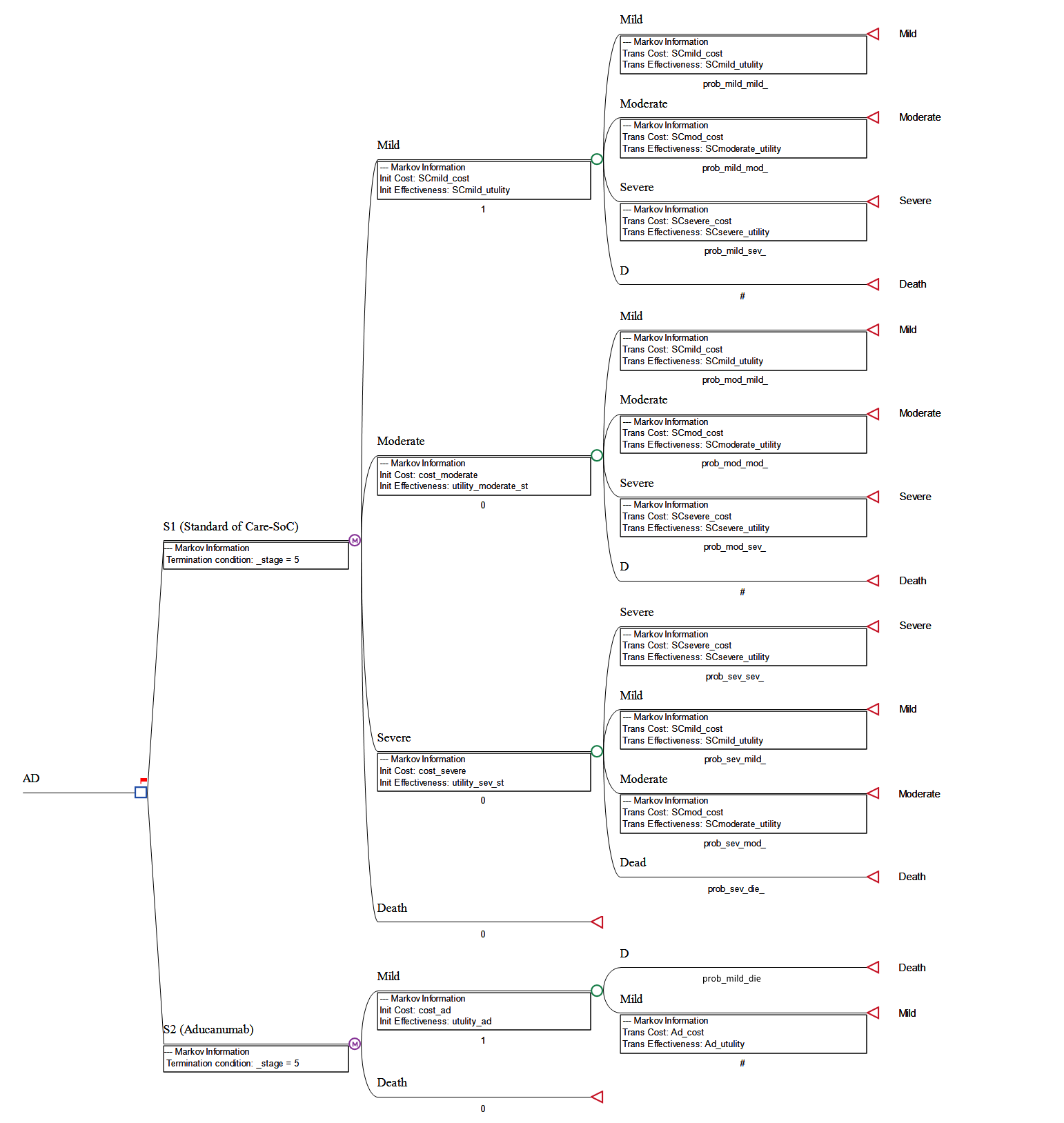


**Figure 2.** Acceptability of Aducanumab at Different Thresholds


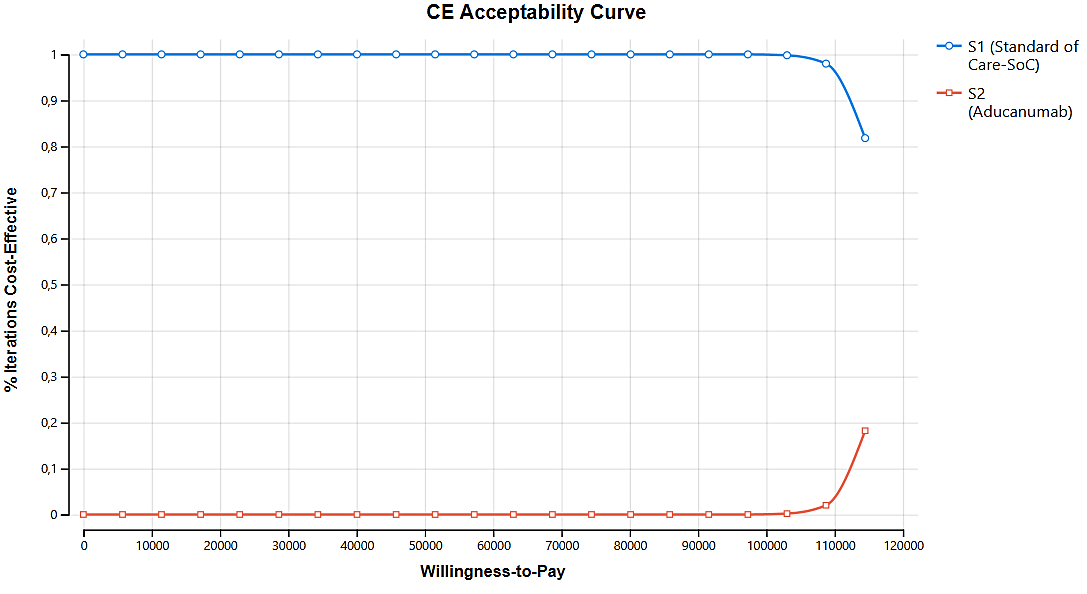

Supplement: Supplementary file 1 — Additional file 1: Figure S1. Creation of Markov Model in Treeage Program. Figure S2. Acceptability of Aducanumab at Different Thresholds. [file 12962_2023_463_MOESM1_ESM.docx]
